# Bromoderma in an infant*

**DOI:** 10.1590/abd1806-4841.20165013

**Published:** 2016

**Authors:** Isadora da Rosa Hoefel, Fernanda Oliveira Camozzato, Laura Netto Hagemann, Deise Louise Bohn Rhoden, Ana Elisa Kiszewski

**Affiliations:** 1 Universidade Federal de Ciências da Saúde de Porto Alegre (UFCSPA) - Porto Alegre (RS), Brazil; 2 Private clinic - Porto Alegre (RS), Brazil; 3 Private clinic - Caxias do Sul (RS), Brazil; 4 Hospital Nossa Senhora das Graças - Canoas (RS), Brazil

**Keywords:** Acneiform eruptions, Drug Eruption, Infant

## Abstract

Bromoderma is a cutaneous eruption caused by the absorption of bromide. Clinical manifestations include acneiform and vegetative lesions. We report the case of an infant with bromoderma caused by the use of syrup for abdominal colic containing calcium bromide. The lesions regressed after discontinuation of the drug.

## INTRODUCTION

Bromoderma is characterized by cutaneous eruptions caused by the ingestion, inhalation or direct contact with bromides.^1^ Bromide is a halogen element widely used in pediatrics in the early 20^th^ century because of its expectorant, sedative, antispasmodic, and especially anticonvulsant effects.^1,2^ Many cases of bromoderma and bromism (systemic bromide poisoning) were reported at that time. However, with the development of new anticonvulsants and the prohibition of the use of solutions containing calcium and potassium bromide in many countries, such cases have become increasingly rare.^2,3^

## CASE REPORT

**Figure 1:**
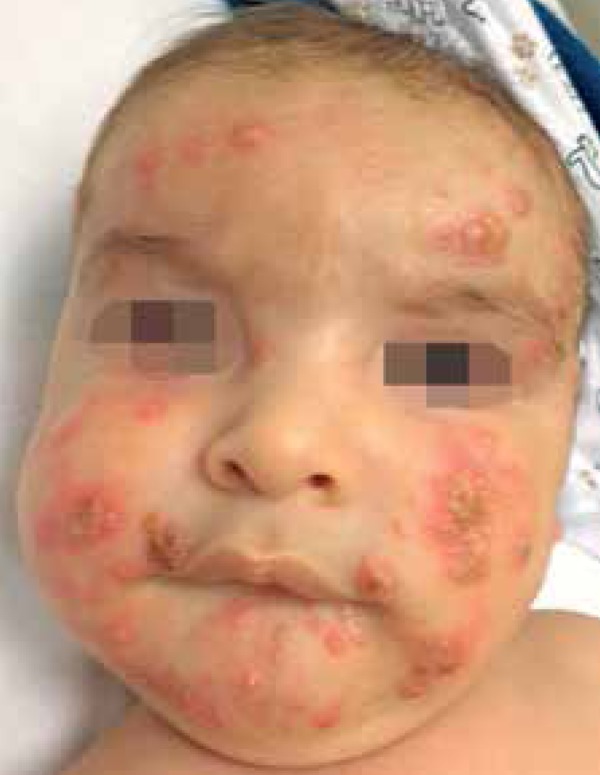
Pustules and crusty areas on the face

**Figure 2:**
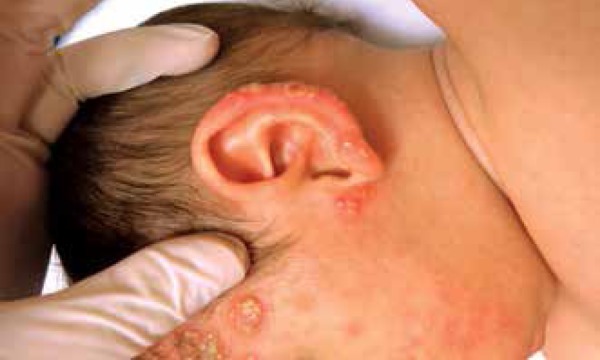
Pustules and crusts on the pinna

**Figure 3:**
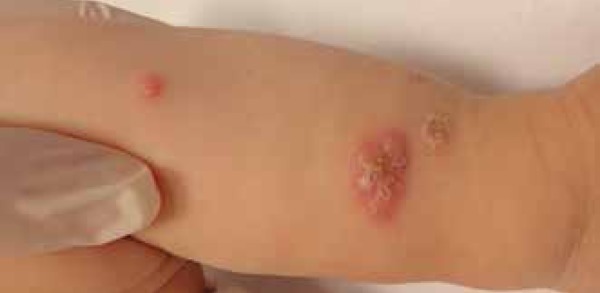
Vegetative plaques formed by coalescence of multiple pustules on the left leg

**Figure 4:**
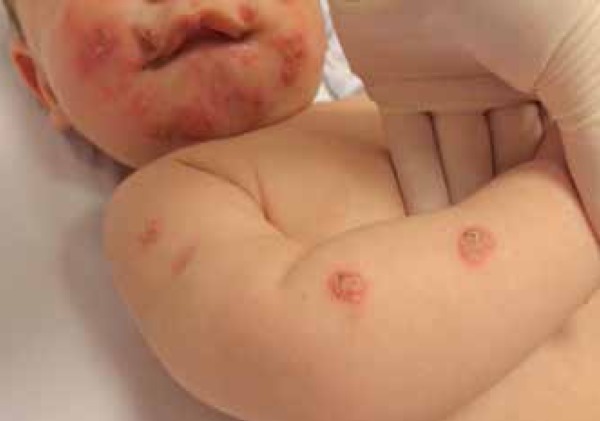
Erythematous papules with pustules inside, following a linear path (sporotrichoid aspect) on the right upper limb

**Figure 5:**
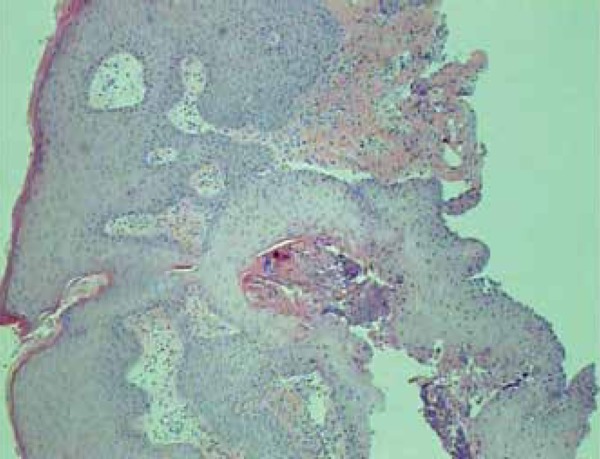
Skin presenting pseudoepitheliomatous acanthosis, keratosis and parakeratosis. Bleeding and eosinophilic and neutrophilic infiltrate in the papillary dermis. Hematoxylin-eosin 45X

**Figure 6:**
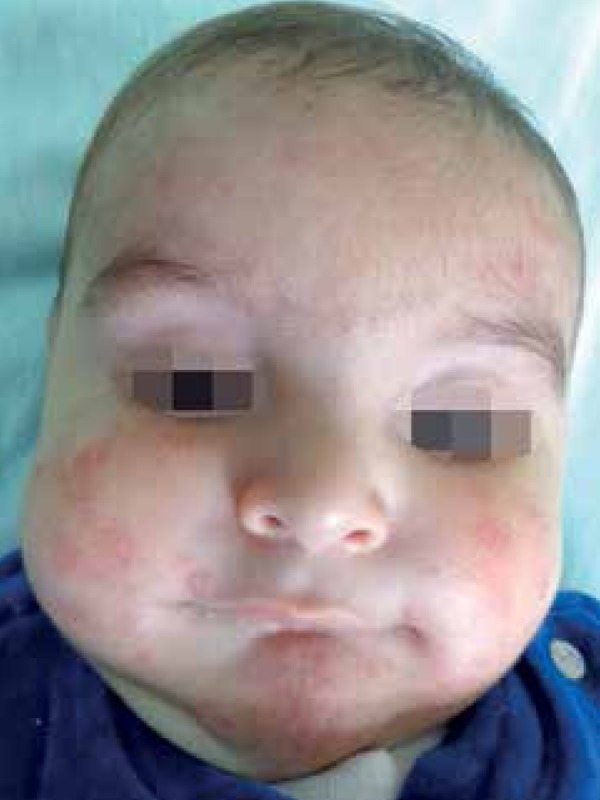
Residual dyschromia and some scars one month after the withdrawal of syrup containing calcium bromide

We report a 5-month-old male infant referred to our institution with a three-month history of skin lesions. Physical examination revealed good general condition and confluent papulopustular lesions forming infiltrating erythematous plaques, some with vegetative aspect. Lesions were on the head and upper and lower limbs. On the head, lesions were spread over the face, scalp and ears (Figures 1 to 4). Physical examination revealed no other changes. The prenatal and perinatal exams were normal. Psychomotor development of the child was also normal. Laboratory tests presented at consultation (blood count, liver and kidney function) were within normal limits. The infant was fed with infant formula. The mother reported that the infant had colic since the first month of life and was receiving daily colic medication and, occasionally, paracetamol. The active ingredient in the colic syrup - a compounded medication - was calcium bromide. Based on clinical findings and on the intake of bromide-containing syrup history, we diagnosed bromoderma. Our diagnosis was later confirmed by pathological examination (Figure 5). Skin lesions regressed slowly in the next 15 days after the drug withdrawal and disappeared in 30 days (Figure 6).

## DISCUSSION

Although the pathogenesis of bromoderma is still unclear, it could be described as a type of delayed hypersensitivity reaction.^1,2^ Lesions usually appear after a prolonged period of use of the medication but can also appear as early as eight days after initial administration. Lesions tend to affect skin areas where there are high concentrations of sebaceous glands.^4^ Diagnosis can be made based on skin lesions, bromide intake history and healing of lesions after discontinuation of the drug.^1^

Studies reveal that systemic poisoning occurs in 1-10% of patients receiving bromide. However, it is believed that the occurrence of skin lesions is not dose-dependent. Three theories are proposed: the toxic theory, which supports the idea that the elimination of bromine by the eccrine and sebaceous glands produce an intense inflammatory reaction; the biotrophic theory, which proposes that bromide could increase the pathogenicity of saprophytic skin germs; and finally, the immunological theory, which suggests that halogenated compounds produce a hypersensitive reaction - the most accepted theory today.^1^

Clinical manifestations can be mild - acneiform rash with papules and pustules - or more pronounced - such as panniculitis, ulcers and vegetative nodules, known as tuberous bromoderma or vegetating bromoderma. Typically, lesions involve the face, scalp and lower limbs.^5,6^ Histopathological findings that support the diagnosis include pseudoepitheliomatous hyperplasia, intraepidermal abscesses and perifollicular and periadnexal neutrophilic infiltrates. Bromoderma diagnosis can be performed even with normal serum bromide levels since bromism is not usually present.

Differential diagnoses are deep mycoses (mainly sporotrichosis, cryptococcosis and cromomycosis), pyoderma, acne, pyoderma gangrenosum, vegetating pemphigus, Sweet's syndrome, eosinophilic folliculitis, atypical mycobacterial infections, cutaneous tuberculosis and congenital syphilis.^2,3,4^

The main treatment is the suspension of bromide intake. Secondary therapies are not in absolute consensus. As the half-life of bromide in the human body is around 10-14 days, excretion is performed through the kidneys. Measures to stimulate this excretion - such as intense hydration and use of diuretics and mannitol - are also suggested.^2,4,5^
